# Supplementary material for: Distinctive Features of Extracellular Vesicles Present in the Gastric Juice of Patients with Gastric Cancer and Healthy Subjects
Source: Int J Mol Sci. 2025 Jun 18;26(12):5857. doi: 10.3390/ijms26125857 (PMC12193056; doi:10.3390/ijms26125857)
Supplement: Supplementary file 1 [file ijms-26-05857-s001.zip › Supplementary Figure S1.pdf]

Supplementary Figure S1. Cryo-EM images illustrating the morphological diversity of GJ EVs isolated from GC patient.

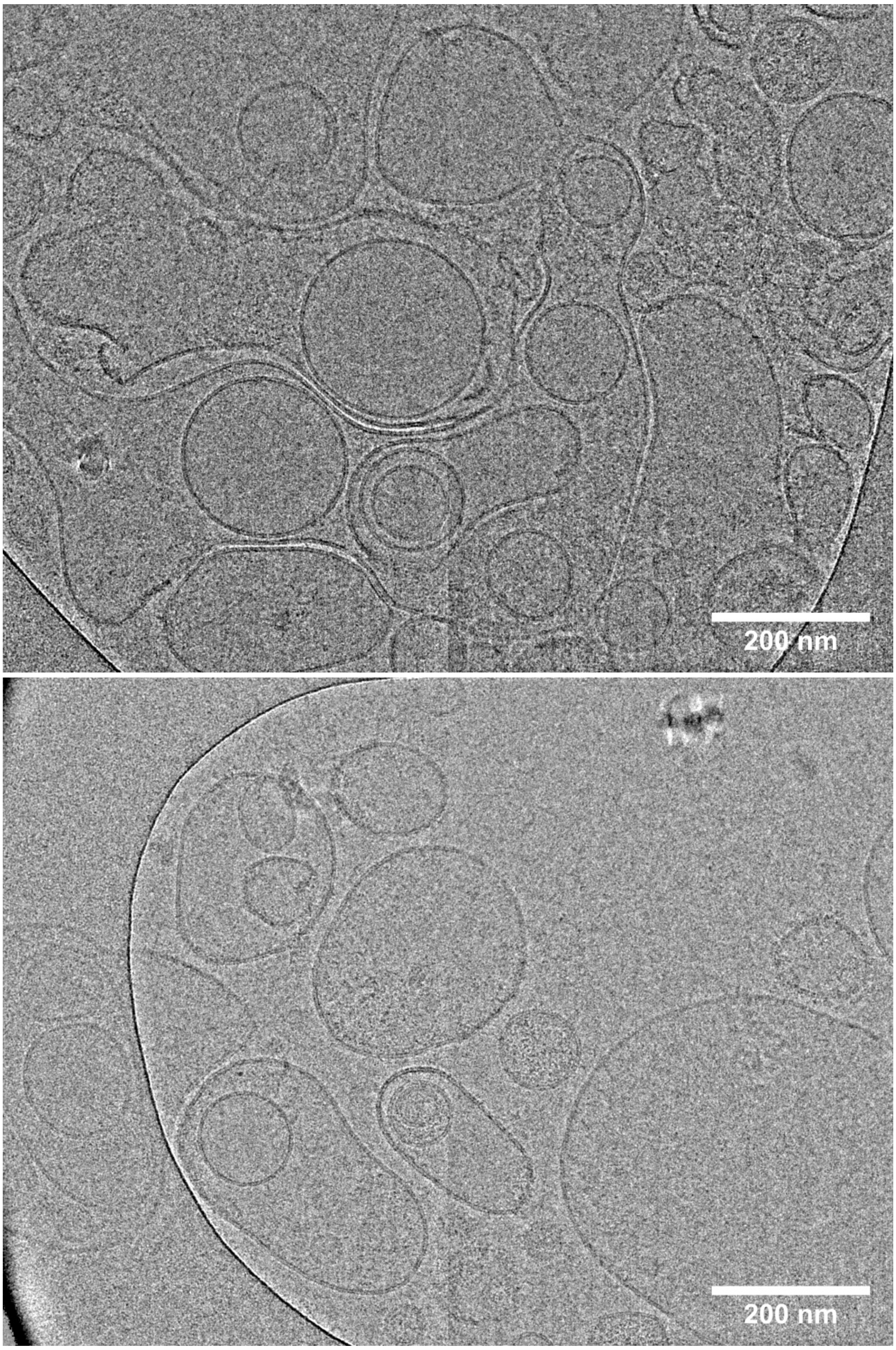

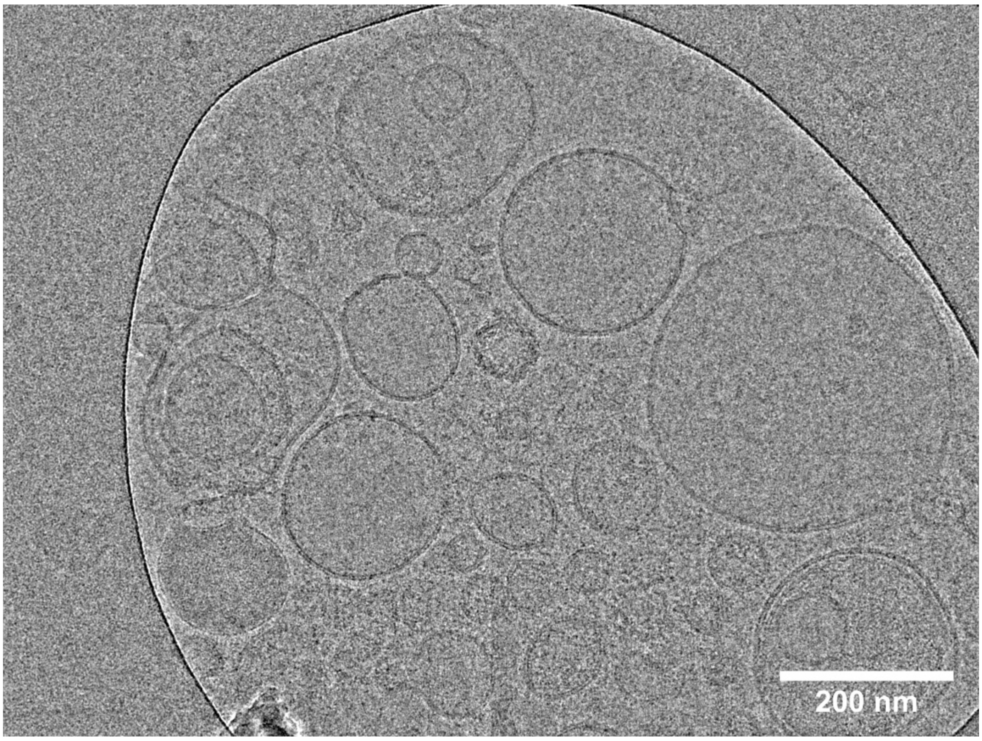
200 nm

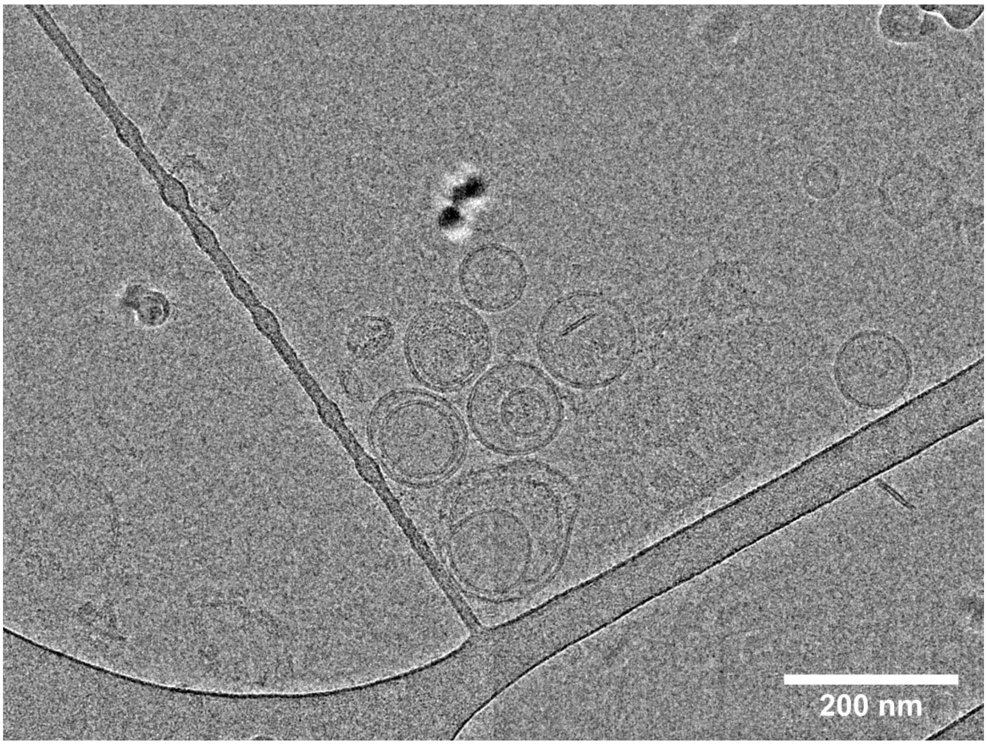
200 nm

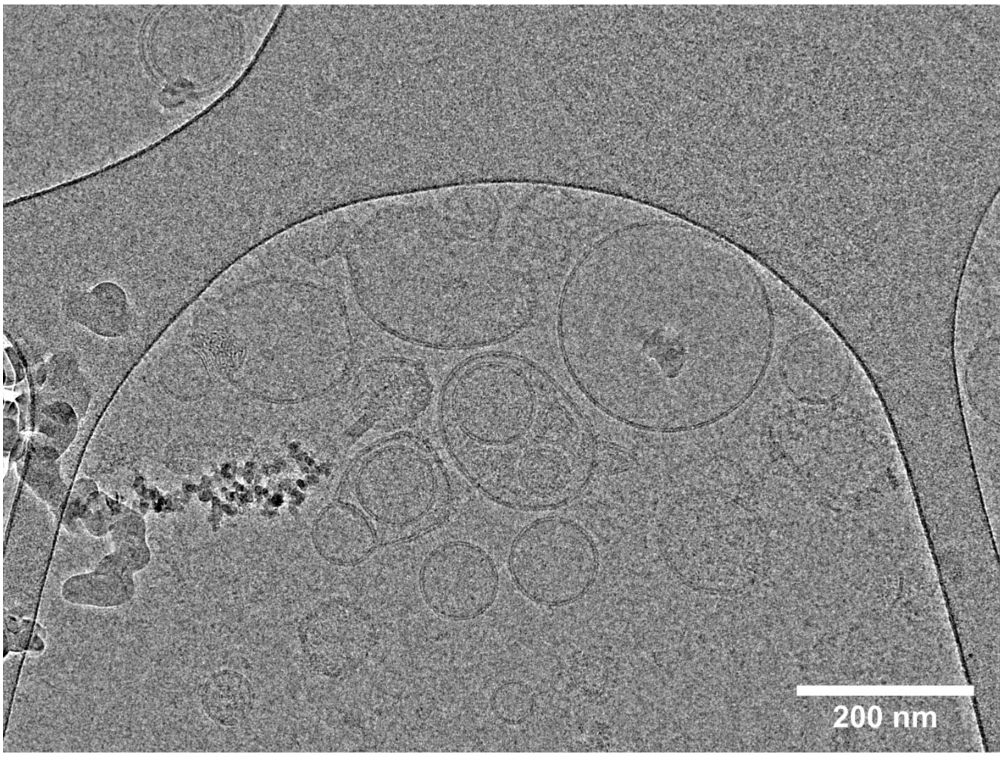
200 nm

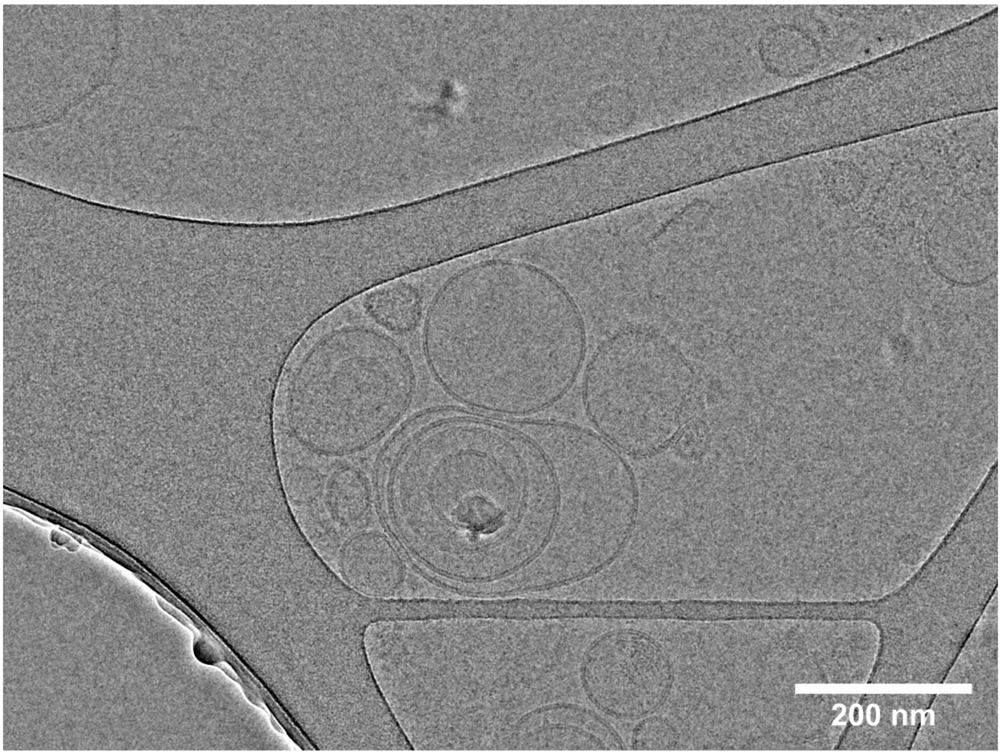
200 nm
