# Supplementary material for: Distinctive Features of Extracellular Vesicles Present in the Gastric Juice of Patients with Gastric Cancer and Healthy Subjects
Source: Int J Mol Sci. 2025 Jun 18;26(12):5857. doi: 10.3390/ijms26125857 (PMC12193056; doi:10.3390/ijms26125857)

Supplementary Figure S2. Analysis of CD9 expression and vesicle size across gastric cancer stages. (A) Distribution of CD9-positive and CD9-negative gastric juice samples across disease stages (I–IV), shown as absolute counts. (B) Proportion of CD9-positive samples by stage. (C) Boxplot of average EV size (measured by NTA) in samples from different stages.

A

Stage	CD9(+)	CD9(−)	Overall
**I**	2	2	4
**II**	1	2	3
**III**	8	7	15
**IV**	12	6	18
**Overall**	23	17	40

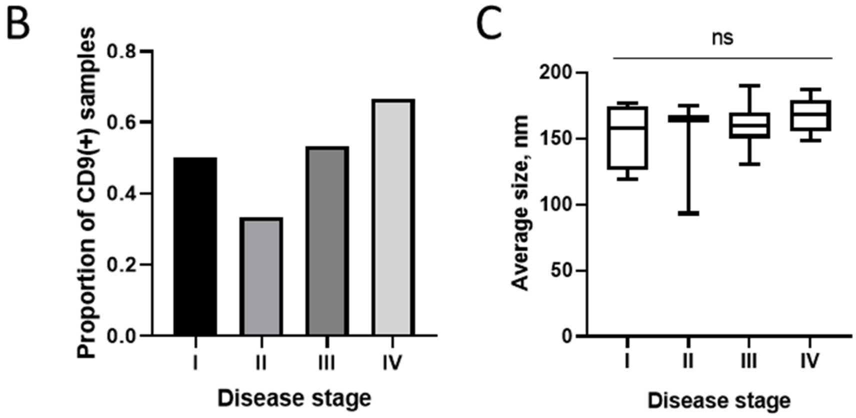

Supplement: Supplementary file 1 [file ijms-26-05857-s001.zip › Supplementary Figure S2.pdf]
